# Platinum Derivatives Effects on Anticancer Immune Response

**DOI:** 10.3390/biom10010013

**Published:** 2019-12-20

**Authors:** Cédric Rébé, Lucie Demontoux, Thomas Pilot, François Ghiringhelli

**Affiliations:** 1 Platform of Transfer in Cancer Biology, Centre Georges-François Leclerc, F-21000 Dijon, France; 2 University of Bourgogne-Franche-Comté, F-21000 Dijon, France; lucie.demontoux@hotmail.fr (L.D.); tpilot@cgfl.fr (T.P.); fghiringhelli@cgfl.fr (F.G.); 3 INSERM LNC-UMR1231, F-21000 Dijon, France

**Keywords:** oxaliplatin, cisplatin, carboplatin, immunogenic cell death, immune checkpoints

## Abstract

Along with surgery and radiotherapy, chemotherapeutic agents belong to the therapeutic arsenal in cancer treatment. In addition to their direct cytotoxic effects, these agents also impact the host immune system, which might enhance or counteract their antitumor activity. The platinum derivative compounds family, mainly composed of carboplatin, cisplatin and oxaliplatin, belongs to the chemotherapeutical arsenal used in numerous cancer types. Here, we will focus on the effects of these molecules on antitumor immune response. These compounds can induce or not immunogenic cell death (ICD), and some strategies have been found to induce or further enhance it. They also regulate immune cells’ fate. Platinum derivatives can lead to their activation. Additionally, they can also dampen immune cells by selective killing or inhibiting their activity, particularly by modulating immune checkpoints’ expression.

## 1. Introduction

Genetic instability allows neoplasia cell growth. The phenotype of these cells was described by Hanahan and Weinberg, and it is characterized by sustained proliferative signaling, replicative immortality, resistance to cell death, metabolic reprogramming, promotion of angiogenesis and inflammation, activation of invasion and metastases formation, insensitivity to anti-proliferative factors, avoidance of immune suppression, and development of an immunosuppressive environment [1].

This model shows the importance of the relationship between cancer cells and host. Tumor environment, particularly the immune system, plays a major role in the development and elimination of cancer cells. Understanding the effects of tumor cells and antitumor treatments, such as radiotherapy and chemotherapy, on the immune system will help the development of support treatments, improving patients’ survival. Among chemotherapeutic drugs, platinum derivatives have a huge importance in the therapeutic arsenal. Oxaliplatin, cisplatin and carboplatin are commonly used for the treatment of colorectal, lung, ovarian, head and neck, breast, bladder and testicular cancers [2]. However, knowledge on the immune environment of each tumor type and on the effects of platinum derivatives on immune cells might explain the differences observed in platinum derivatives’ effectiveness according to cancer types.

Here, we will analyze the effects of platinum derivatives on immunogenic cell death (ICD), immune cells and immune checkpoints.

## 2. Antitumor Immune Response after Chemotherapy Treatments

Cell death is characterized by morphological alterations that have been historically used by *The Nomenclature Committee on Cell Death* (*NCCD*) to classify this cellular process into three different forms: type I or apoptosis, type II or autophagy, and type III or necrosis. Although limited, this morphological classification remains widely used, independently of essential molecular aspects of the cell death process. New NCCD classifications focus on molecular mechanisms and on the biochemistry of intracellular signalization effectors. Currently, it is clear that connections and overlaps exist between the different types of cell death. The majority of chemotherapeutic drugs are known to induce apoptosis or necrosis. Engagement of a particular type of cell death depends on the stress type, interaction between induction factors, genetic cell background, organelle content and enzymatic proteins arsenal [3,4].

The action of chemotherapy relies not only on its cytotoxic effect on cancer cells, but also on its ability to affect immune cells.

### 2.1. Immunogenic Cell Death (ICD)

Some chemotherapeutic drugs can trigger damage, leading to protein expression at the cell surface, cytokine secretion, or plasma membrane rupture and release of the intracellular content. Released cytoplasmic molecules are danger signals, called DAMPs (damage-associated molecular patterns), which enable the sensitization of tumor antigen recognition by the immune system.

Guido Kroemer’s team was the first to highlight the capacity of some chemotherapeutic drugs to induce ICD, characterized by an ability to activate an adaptive immune response specific to exogenous (virus) and endogenous (tumor cells) antigens expressed by dying cells. Murine colon tumor cells (CT26) treated in vitro by lethal concentrations of chemotherapy (such as doxorubicin) enable the vaccination of syngeneic mice against further injection of tumor cells, without any adjuvant. These effects are not reproduced in immunodeficient mice. These studies evidence that tumor cells are able to establish an immune response and require two different factors, antigen presentation and innate immunity stimulation by DAMPs [5]. Thus, a high number of chemotherapeutic (mitoxantrone, oxaliplatin) or physical agents (high pressure, radiation) are able to induce ICD. These inductors can trigger an apoptotic caspase-dependent cell death and endoplasmic reticulum (ER) stress, moving the lines that previously considered apoptosis as a non-inflammatory and non-immunogenic cell death [6].

**Figure 1:**
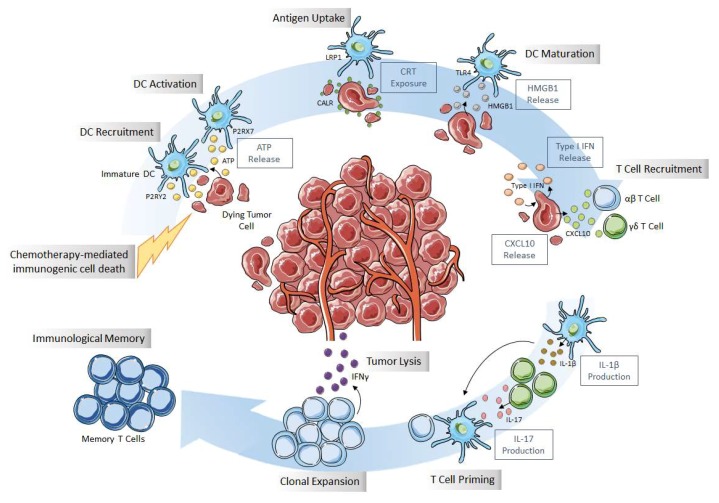
Immunogenic cell death (ICD) characteristics. In response to ICD inducers, cancer cells secrete ATP (which leads to dendritic cell (DC) recruitment and activation), express calreticulin (CRT) and other endoplasmic reticulum (ER) chaperone molecules at the plasma membrane, and release high-mobility group box 1 (HMGB1), leading to DC maturation and initiation of intrinsic type I interferon (IFN) response and CXC-chemokine ligand 10 (CXCL10) production promoting T-cell recruitment. Acting as DAMPs (damage-associated molecular patterns), these molecules favor phagocytosis of cellular debris by antigen-presenting cells (APCs) such as DCs. All these events will engage an adaptive immune response, implicating αβ and γδ T cells and the establishment of an immunological memory. Such a response is able to eradicate cancer cells through an IFNγ-dependent mechanism. Adapted from Galluzzi et al. [7].

ICD is mainly characterized by calreticulin (CRT) exposure at the cell surface of dying tumor cells, release of ATP and HMGB1 (high-mobility group box 1) in the extracellular space, and subsequent activation of an intrinsic IFNα (interferon) pathway and CXCL10 (CXC-chemokine ligand 10) release (Figure 1) [7].

Some chemotherapeutic agents were shown to be able to induce all these features, such as mitoxantrone, doxorubicin, epirubicin, bleomycin or cyclophosphamide [8]. However, this should be considered with caution as these observations can depend on the cell type, concentration and time of exposure to chemotherapy. This will be detailed below for platinum derivatives, but it can also be true for other compounds. Therefore, arose the concept of combining chemotherapies or using new compounds to facilitate drug-induced ICD, mainly by activating initial events responsible for ICD marker appearance. One classical example is CRT exposure, which can be stimulated through ER stress induction.

### 2.2. ER Stress and CRT Exposure

Immunogenic chemotherapy induces CRT exposure at the cell surface of dying cells, which will behave as an “eat me” signal for dendritic cells (DCs). CRT is a chaperon protein that fixes calcium at the ER level. Its translocation from the ER to the cell surface occurs prematurely, before the transition of phosphatidylserines on the external layer of the plasma membrane (a characteristic event of apoptosis) [9]. Three signaling steps are required to lead to CRT exposure: ER stress with eIF2α (eukaryotic initiation factor 2α) phosphorylation, an apoptotic signal, and a transport step from the ER to the Golgi apparatus. This process begins with a high production of radical oxygen species that may allow the activation of ER stress. PERK (protein kinase RNA-like endoplasmic reticulum kinase) phosphorylates eIF2α, which favors the apoptotic process by inducing caspase-8 activation and consequently the cleavage of its target Bap31. Cleaved Bap31 entails Bax and Bak oligomerization, leading to the disruption of mitochondrial permeability, resulting in cytochrome c release in the cytosol. The association of two steps (ER stress and apoptosis) modulates vesicular transport, allowing CRT exocytosis through its translocation from the ER to the plasma membrane via the Golgi apparatus. This transport is dependent on VAMP1 (vesicle-associated membrane protein 1) and SNAP25 (synaptosomal-associated protein 25) [6]. Recognition of membrane-associated CRT by CD91 and CD69 on APCs (antigen-presenting cells—DCs, macrophages, neutrophils) favors the phagocytosis of apoptotic bodies and is required to initiate adaptive immune response [10]. Moreover, CRT expression on patient tumor cells is a prognosis marker [11].

### 2.3. ATP Release

Many ICD inducers (doxorubicin, mitoxantrone, oxaliplatin) can trigger ATP release from dying tumor cells. Even if this release can be induced by several mechanisms, it seems that the main pathway involved is autophagy, which enables high levels of intracellular ATP. ATP included in cytoplasmic vesicles is transferred into autolysosomes through an autophagy process dependent on ATG5 (autophagy-related gene 5), ATG7 and Beclin1. Autolysosomal LAMP1 (lysosomal-associated membrane protein 1) favors lysosome membrane rupture and ATP release in the cytosol or in the extracellular environment if autolysosomes merge with the plasma membrane. ATP can also be released outside of the cell by the membrane channel pannexin 1 after activation by caspases [12,13]. Extracellular ATP plays an attractant role on immune cells and can also activate inflammasome. The fixation of ATP on its receptor P2Y2 on monocytes or DCs induces their recruitment and differentiation at the tumor site. Once recruited and activated by “eat me” signals in the tumor, naive immune cells need further activation signals to improve their antitumor capacities. ATP signaling via P2RX7 leads to the release of potassium ions and to NLRP3 inflammasome activation in DCs and macrophages. The assembly of NLRP3 inflammasome triggers caspase-1 activation, which in turn cleaves and maturates IL-1β and IL-18, two pro-inflammatory cytokines [14]. Although P2RX7 is expressed on many cell types, ATP mainly acts on DCs after ICD. Overexpression of ectonucleotidases (CD39 and CD73 responsible for ATP degradation into AMP and immunosuppressive adenosine) on tumor cells, T lymphocytes or regulatory T-cells surface limits ATP-mediated immunosurveillance and stops immune response [15,16].

### 2.4. HMGB1 Release

HMGB1 is a ubiquitous protein playing an important role in nucleosome stability, transcriptional regulation and DNA repair. In addition to its nuclear function, HMGB1 takes part in inflammation, cell differentiation and migration, and tumor metastasis of remaining cells after chemotherapy [17]. HMGB1 is secreted by macrophages [18], DCs [19] and natural killer (NK) cells [20] in response to infection or injury. After chemotherapy treatments, HMGB1 is released by dying tumor cells after nuclear and cytoplasmic membranes’ permeabilization. This is a late event in the ICD process. However, the mechanism responsible for HMGB1 release remains to be clearly elucidated. Once in the intercellular space, HMGB1 binds toll-like receptors (TLR) 2 and 4 or RAGE (receptor for advanced glycation end products) [21,22]. Binding of HMGB1 to DCs’ TLR4 seems to be the signal-enabling perception of ICD. This restricts lysosomal degradation of phagocyted material, then leading to antigen presentation. In contrast, RAGE is required for DC maturation. Thus, cell death cannot be seen as immunogenic in the absence of HMGB1 in cancer cells or TLR4 in myeloid cells [4]. In addition, binding of HMGB1 to CXCL2 could allow DCs’ chemotaxis via CXCR4 signaling [23].

Thereby, even if CRT exposure, ATP and HMGB1 release are required, these events separately are not sufficient to allow the immunogenic effect of chemotherapy. Actually, to stimulate the immune system, these three signals are complementary and necessary since they play a role at different levels to attract and activate APCs. ATP and HMGB1 participate in DC recruitment and maturation, whereas CRT favors dying cells’ phagocytosis, allowing tumor peptide presentation and activation of naïve and memory lymphocytes localized in secondary or tertiary lymphoid organs. CD8 and CD4 effector T cells will migrate and infiltrate tumors, inhibiting tumor cell proliferation and leading to tumor cell death through IFNγ, TNFα (tumor necrosis factor α), perforin and granzym (Figure 1) [7].

### 2.5. Secretion of Other Danger Signals

After activation of TLR3 on cancer cells, autocrine and paracrine signaling of type 1 IFN is engaged and will entail the production of CXCL10, a chemotactic factor for T lymphocytes [24]. Other DAMPs can have a role in cancer cell immunogenicity, such as annexin A1, heat shock proteins (HSP70 and 90) release, PDIA3 (protein disulfide isomerase family A member 3) membrane exposure, and lipid metabolites (cardiolipin, formyl peptide) secretion [7].

### 2.6. Methods to Detect ICD Markers

**Figure 2:**
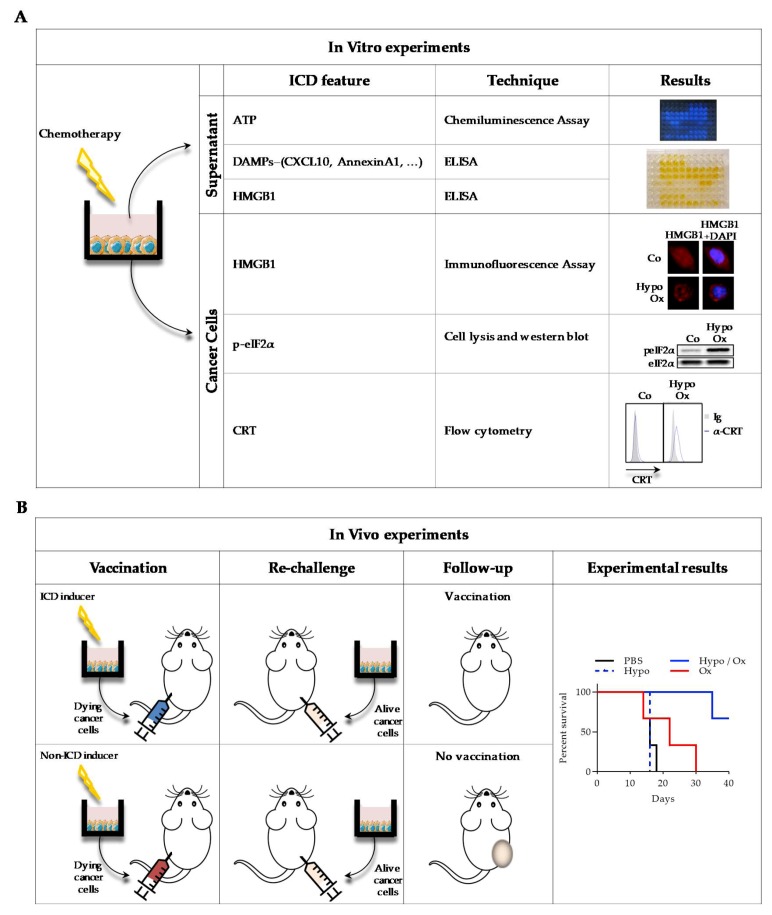
Description of main experimental settings to detect ICD in vitro (**A**) and in vivo (**B**). A: After various chemotherapeutic treatments of cancer cells in vitro, supernatants and cancer cells were recovered. ATP, DAMPs and HMGB1 release was measured in the supernatants, by chemiluminescence assay (ATP) and by ELISA (DAMPs and HMGB1). On the cells, HMGB1 was visualized by immunofluorescence (anti-HMGB1 antibody staining with DAPI (4′,6-diamidino-2-phenylindole)), p-eiF2α (eukaryotic initiation factor 2α) by western blot (anti- p-eiF2α and anti- eiF2α antibodies), and CRT exposure by flow cytometry (anti-CRT antibody with DAPI). To obtain results illustration, CT26 murine colon cancer cells were treated or not (Co) for 30 min with 400 µM oxaliplatin in hypotonic medium (Glucose 2.5%) and left for 24 h in a new medium before experiments. B: Mice were subcutaneously (s.c.) injected with CT26 previously treated in vitro with chemotherapy. One week later, live cells were s.c. injected on the opposite flank, and tumor growth was monitored. When chemotherapy is an ICD inducer, no tumor is detected, while if it is not, a tumor will grow at the secondary site of injection. To obtain results illustration, mice were s.c. injected with 500,000 CT26 cells treated as indicated in vitro. One week later, mice were s.c. injected in the opposite flank with 500,000 live CT26 cells. Survival (n = 6 animals per group) was represented using the Kaplan–Meier method. *p* < 0.01, using log-rank test.

Several assays are available to monitor ICD features (Figure 2). To detect these markers, classical assays have been commercialized, such as ELISA kits or antibodies. Laboratories are required to have common apparatuses such as chemiluminescence and OD readers, western blot and real-time qPCR equipment, fluorescence microscope and flow cytometer.

CRT exposure at the cell surface can be visualized by flow cytometry. To detect this feature, non-permeabilized cells are stained with a specific anti-CRT antibody and with DAPI (4′,6-diamidino-2-phenylindole) or propidium iodide. Because anti-CRT can recognize both extra and intra-cellular CRT, it is imperative to remove DAPI^+^ or PI^+^ cells (corresponding to cells with permeabilized membranes) to be sure to detect only externalized CRT [9,25,26]. After staining, cells can also be observed under a microscope. Another method consists in biotinylation of cell surface proteins, which can be precipitated using streptavidin and analyzed by western blot, using an anti-CRT antibody [9]. One difficulty of this method is the need to use pre-apoptotic cells with intact membranes to avoid false-positive results with intra-cellular protein detection. Moreover, genetically modified cells can be used, such as CRT- HaloTag™ [27] or CRT- GFP [28] transfected cells.

ER stress is responsible for CRT translocation from the ER to the cell membrane. So, an indirect way to evaluate this phenomenon is to analyze ER stress response, such as eIF2α phosphorylation by western blot [25], XBP1 (X-box binding protein 1) mRNA splicing by real-time qPCR [29], or ATF6 (activating transcription factor 6) nuclear translocation by fluorescence microscopy [30].

ATP secretion can be visualized, using the capacity of eukaryotic luciferases to oxidize d-luciferin in an ATP-dependent manner and produce light. Hence, the more ATP is present in the supernatant or in cell lysates, the more light is produced. ATP secretion can be determined by an increase in the supernatant, a decrease in cells, or both [25,31]. Quinacrine (a fluorescent probe which can bind ATP) can also be used to detect intracellular ATP levels by fluorescence microscopy [32].

HMGB1 release in the cell supernatant can be monitored using specific commercialized ELISA kits [25]. Since HMGB1 first translocates from the nucleus to the cytoplasm before release, HMGB1 release can alternatively be assessed by fluorescence microscopy. Using a specific anti-HMGB1 antibody with Hoechst 33342 or DAPI (to stain the nucleus) on chemically permeabilized cells, the loss of nuclear colocalization can be correlated to further HMGB1 release [33]. As for CRT, HMGB1 can be visualized using genetically modified cells expressing HMGB1-GFP [34].

Finally, ICD activation of antitumor immune response can be proved by “vaccination” experiments, consisting in subcutaneous (s.c.) injection of tumor cells previously treated in vitro with chemotherapy in immunocompetent mice. After one week, mice are re-challenged with living cells of the same type, and tumor appearance is monitored at the second injection point. If no tumor grows, mice have been vaccinated and the chemotherapy is considered immunogenic [35].

### 2.7. Antitumor and Protumor Immune Cells

APCs phagocyte antigens in the periphery, migrate to the lymphoid organ, and present processed peptides to T cells. This may drive either priming or tolerance. Several myeloid cell subsets have been described, such as DCs, macrophages, and myeloid-derived suppressor cells (MDSCs) [36]. DCs are the key APCs. DCs are immune sentinels and may trigger a T-cell response against microbial pathogens, inflammation and tumors [37,38]. Tumor-associated macrophages (TAMs) are generally classified into two subsets, M1 and M2 macrophages. M1 express nitric oxide synthase, produce TNF-α and IL-12, have potent anti-microbial properties, and promote Th1 responses. M2 produce arginase-1, TGF-β, and IL-10, and support Th2-associated effector functions [39,40].

MDSCs are immature myeloid cells, which suppress T-cell activation [41]. A high number of MDSCs was found in the blood of patients with different types of cancers [42,43]. In humans and mice, MDSCs from tumor bearers suppress antitumor immunity mainly by inhibiting antigen-specific major histocompatibility complex (MHC) class I-mediated CD8^+^ T-cells activation [44]. Generally, MDSCs are divided into PMN-MDSCs (polymorphonuclear MDSCs), sharing phenotypic and morphologic features with neutrophils, and M-MDSCs (monocytic MDSCs), similar to monocytes [45].

T lymphocytes participate in host innate anticancer immune response [46]. Clinical outcomes and survival in many types of cancers, such as breast [47], colorectal [48] and lung cancers [49], are associated with tumor-infiltrating CD4^+^ and CD8^+^ T cells. CD4^+^ T helper (Th) cells support hematopoietic cells, such as cytotoxic CD8^+^ T lymphocytes (CTLs), NK cells, DCs and macrophages, in immune processes. CD4^+^ Th cells recognize specific antigen peptidic sequences, allowing their activation. These peptides are presented to CD4^+^ Th cells by APCs, through MHC class II molecules. After activation, CD4^+^ Th cells rapidly proliferate and secrete cytokines that will assist or inhibit the immune response [50]. The ability of CD8^+^ T lymphocytes to kill malignant cells is induced after recognition of specific antigenic peptides by the TCR (T cell receptor). These peptides are presented on the surface of target cells by human leukocyte antigen class I (HLA-I)/beta-2-microglobulin (β2m) complexes. These functions can be mediated directly, through exocytosis of cytotoxic granules containing granzym and perforin into the target cells or indirectly, through secretion of cytokines, including TNF and IFN-γ, both resulting in cancer cell destruction [51].

### 2.8. Checkpoint Inhibitors

Immune checkpoints negatively control T-cell immunity. The discovery of inhibitors of these checkpoints has opened new clinical opportunities for cancer immunotherapy. The most studied are programmed death receptor 1 (PD-1) and cytotoxic T lymphocyte antigen 4 (CTLA-4). PD-1 is mainly expressed on activated CD8^+^ T cells, Treg and Tfh localized in the tumor microenvironment, but also on NK and activated B cells. The fixation of PD-1 ligands PD-L1 (B7-H1) and PD-L2 (B7-DC) on PD-1 participates in T-cell exhaustion [52]. PD-1 activation triggers the inhibition of the phosphatidylinositol 3-kinase (PI3K)/Akt pathway, leading to the suppression of survival and proliferation of T cells, a decrease in protein synthesis, and interleukin (IL)-2 release [53]. Directly targeting the receptor with pembrolizumab or nivolumab has shown promising effects in melanoma. PD-L2 seems to have a low importance, since targeting PD-L1 showed beneficial effects in some early phase clinical studies, raising the question about the importance of PD-1/PD-L2 interaction [54]. This can be partly explained by the fact that PD-L1 can also interact with the co-stimulatory molecule B7, thus inhibiting T cells [55]. CTLA-4 is expressed both on T helper and Treg cells, and binds its ligands CD80 (B7–1) and CD86 (B7–2) present on APCs [52]. CD80/CD86 expressed on DCs triggered CTLA-4 signaling, inducing the release of IDO (indoleamine 2,3-dioxygenase), an enzyme that degrades L-tryptophan leading to an arrest of T cells’ growth [56]. Ipilumimab, an anti-CTLA-4 in association or not with chemotherapy, has shown an improved overall survival in patients suffering from prostate, non-small cell lung cancers (NSCLCs) or melanoma [54]. Even if patients benefit from the use of such antibodies, treatments must be improved by optimizing the dosage, by thinking about possible associations with chemotherapy and radiotherapy, or by controlling immune-related events, for example [54].

Other checkpoints can also be involved in tumor immune escape such as TIM-3 (T-cell immunoglobulin and mucin domain-containing 3), LAG-3 (lymphocyte activation gene 3 protein), IDO1, BTLA (B- and T-lymphocyte attenuators), A2A-R (A2A adenosine receptor) and VISTA (V-domain immunoglobulin suppressor of T-cell activation) [52,57].

## 3. Main Platinum Derivatives Used in Cancer Treatment

Cisplatin, carboplatin and oxaliplatin, the main platinum derivatives used in clinic, share similarities in their structure but also have differences in their mode of action (transport or DNA modifications for example) and are not used for the treatment of the same types of cancers (Table 1).

### 3.1. Cisplatin

Cisplatin was also known as Peyrone’s chloride, from Michele Peyrone, the researcher that originally synthesized it in 1884. Its anticancer properties were evidenced in the 1970s, and it was approved by the FDA (federal drug administration) in 1978. Cisplatin has been used to treat numerous solid neoplasms, such as ovary, testis, bladder, colon, rectum, lung, or head and neck cancers [66].

Cisplatin is an alkylated agent that mainly has an effect on double -helix DNA (on nitrogen 7 of guanine or adenosine) by forming intra- and inter-strand connections [60]. These adducts form between two puric adjacent bases (between two guanines or between guanine and adenosine) localized on the same strand or on different strands, and lead to DNA structure distortion (47°). Replication and transcription are disrupted, and DNA-cisplatin adducts are recognized by many DNA repair complexes, mainly NER (nucleotide excision repair) and MMR (mismatch repair) to avoid damage, or if this is not possible, to entail apoptotic cell death [64,65].

Only 1% of intracellular cisplatin can bind DNA. Indeed, cisplatin can also recognize a lot of substrates in the cytoplasm, such as gluthatione, methionine, metallothionein and other proteins, through their cysteine residues. Cisplatin displays an important cytotoxic activity by tipping the redox scale in favor of oxidative stress, leading to mitochondrial membrane permeabilization and DNA damage [64].

Cells incorporate cisplatin using CTR1 (cupper transporter 1), a transmembrane protein implicated in cupper homeostasis [58]. Other cupper transporters, Type P ATPases ATP7A and ATP7B, also participate in cisplatin import. ATP7A keeps cisplatin in vesicular structures, impairing its spreading, whereas ATP7B is responsible for cisplatin efflux. Some channels seem to be involved in cisplatin transport, namely Na^+^K^+^ ATP pumps and VRAC (volume-regulated anion channels). A deregulation of these transporters is partly responsible for cisplatin resistance phenomena developed by some cancer cells. A reduced incorporation, an increased efflux or a strengthened cytoplasmic confinement/degradation lead to cancer cell resistance and to the establishment of an adaptive response [60]. Worth noting, cisplatin has numerous acute and late side effects such as nausea, vomiting, nephrotoxicity, myelosuppression (thrombocytopenia, leucopenia, anemia) and peripheral sensory neuropathy (ototoxicity), which will limit treatment dosage [64].

Following cisplatin discovery, 23 other drugs containing platinum were tested in clinical trials, but only two (oxaliplatin and carboplatin) obtained approval for wide international commercialization, while three others (nedaplatin, lobaplatin and heptaplatin) were only authorized in a limited number of countries [70].

### 3.2. Carboplatin

Carboplatin was developed to reduce the toxicity observed with cisplatin, and was put on the market in 1989. The mechanism of action of carboplatin is similar to cisplatin. However, it forms fewer intra-strand and, less frequently, inter-strand connections than cisplatin at equimolar concentrations [63]. Thus, DNA-carboplatin adducts are recognized by many DNA repair complexes, mainly NER and MMR [65]. The similarities between cisplatin and carboplatin also lead to similar transport mechanisms, i.e., CTR1 and ATP7B [58,60]. Due to its lower reactivity, the neurotoxicity and ototoxicity of carboplatin are less important. This allows the use of higher and more aggressive dosages when compared to cisplatin. However, the dosage is limited due to serious myelosuppression. Notably, a strong thrombocytopenia is observed, together with neutropenia and anemia [60,66]. Carboplatin is mainly used in ovary, lung and ENT (ear, nose and throat) cancers but showed reduced efficacy in testis, bladder and epidermoid head and neck cancers. Cancer cells present resistance to carboplatin with similar mechanisms to those observed for cisplatin, raising similar questions for clinicians [67]. Consequently, cisplatin remains more often used to treat these particular cancer types.

### 3.3. Oxaliplatin

Oxaliplatin is a third-generation platinum analogue, which obtained its FDA commercialization authorization in 2002 for the treatment of colorectal cancers. It is one of the most widely used, alone or in combination with others, chemotherapeutic drugs to treat stage II/III colon cancers, metastatic colorectal cancers and NSCLCs (non-small cell lung cancers) [68].

This drug is composed of a platinum atom associated with oxalate and a ligand, DACH (diaminocyclohexane). Oxalate significantly reduces oxaliplatin reactivity and consequently limits its toxicity, such as peripheral sensory neuropathy [60]. The DACH ligand plays a major role in cytotoxicity and avoids cross-resistance with cisplatin. DACH lipophilic characteristics increase passive absorption of oxaliplatin when compared to cisplatin and carboplatin [61]. This explains why oxaliplatin uses other cellular incorporation pathways such as organic cation transporters (OCT) 1 and OCT2. Overexpression of these transporters significantly increases oxaliplatin cellular accumulation (with no effect on cisplatin and carboplatin). Colorectal cancer cells express high levels of OCT transporters, and this may explain why oxaliplatin is efficient in these types of cancers. The role of CTR1 in the transport of oxaliplatin is less evident than for cisplatin. However, resistance acquisition is correlated with a reduced expression of CTR1. A lower β1 subunit of Na^+^, K^+^ -ATPase expression is also observed in oxaliplatin-resistant cancer cells. Copper efflux transporters also seem to play a major role in oxaliplatin sensitivity [60].

Similarly to cisplatin, oxaliplatin essentially forms crosslinks between adjacent guanines or, to a lesser extent, between guanine and adenine. However, oxaliplatin-DNA adducts are more efficient at inhibiting DNA synthesis. The voluminous size and lipophilic properties of the DACH ligand allow oxaliplatin to induce DNA conformational distortions and to drive a specific recognition of DNA-oxaliplatin adducts. These adducts are not classically recognized by MMR, rendering oxaliplatin cytotoxicity independent of this type of DNA repair. Moreover, increased DNA-protein crosslink, i.e., DNA synthesis bypassing platin-DNA adducts, is not correlated with oxaliplatin cytotoxicity [60]. Oxaliplatin has a different activity spectrum to cisplatin or carboplatin. It induces apoptosis of cancer cells by triggering Bax oligomerization at the mitochondrial level, allowing cytochrome c release in the cytoplasm [3].

Oxaliplatin is better tolerated than cisplatin and can replace cisplatin in intolerant patients [61]. This chemotherapeutic drug has no hepatic or kidney toxicity but can induce sensorial neuropathy [69].

## 4. Platinum Derivatives and ICD

The main ways to activate antitumor response is to enhance tumor antigen presentation by major histocompatibility complex (MHC) and/or to induce ICD. Cisplatin and oxaliplatin have been shown to stimulate T-cell function, by upregulating MHC class I expression [71,72,73]. However, their ability to induce ICD is less clear.

### 4.1. Direct Effects

Only a few clinically approved agents, including anthracyclines (doxorubicin, idarubicin, epirubicin), alkylating agents (cyclophosphamide), platinum derivatives (oxaliplatin) and anthracenediones (mitoxantrone), have been described to trigger ICD [74]. Guido Kroemer’s team showed that a continuous exposure to oxaliplatin (from 15 to 300 µM, depending on the cell type) induces CRT exposure, ATP and HMGB1 release by different types of cancer cells, such as murine colon cancer (CT26), fibrosarcoma (MCA205) or human bone osteosarcoma (U2OS) [6,31,33,75]. These results were confirmed in human and murine pancreatic tumor cell lines (Panc-1 and Pan02), in murine mammary adenocarcinoma (TSA), in murine glioma cells (KR158) and in murine lung carcinoma (LLC) [76,77,78,79]. In our recent work, we performed in vitro experiments on CT26 murine colon cancer cells with a brief exposure of cancer cells to chemotherapy to reproduce clinical settings and, in particular, hyperthermic intraperitoneal chemotherapy (HIPEC). Under these conditions (30 min exposure and 24 h resting in medium), oxaliplatin had no effect on ICD, contrarily to what has been previously observed [25]. This discrepancy can be explained by the time of exposure. Nevertheless, in metastatic colon cancer patients treated with oxaliplatin-based chemotherapy, the presence of a mutated TLR4 allele is correlated with a significant decrease in progression-free and overall survival [33]. Additionally, oxaliplatin has been shown to induce CXCL10 secretion by melanoma cells, another ICD feature [80].

Concerning cisplatin, CRT exposure, ATP and HMGB1 release can be observed or not, depending on the cell type and/or the concentrations used and/or treatment duration [12,25,31,33,81,82,83]. In LLC, one study reports that 24 h exposure to 20 µM of cisplatin cannot induce immunogenic cell death features [77], whereas in another study, treatment of these cells with 2.5 µM during 24 or 48 h induced ATP release and CRT exposure, but not HMGB1 release [82]. In CT26 cells, cisplatin can induce HMGB1 and ATP release, but not CRT exposure [33,83]. In EG7 cells and U2OS cells, cisplatin is able to induce ATP release, but not CRT exposure [31,81]. In human melanoma cells (BLM), cisplatin induces the three ICD markers [83]. Finally, cisplatin was shown to induce CRT expression in B16 melanoma cells and CXCL10 expression in vivo [84]. However, co-treatment of CT26 or LL/2 tumor-bearing mice by cisplatin with CXCL10 is required to have more important tumor growth inhibition, suggesting that CXCL10 alone is not enough [85].

The ability of carboplatin to induce ICD is less described. One study showed no effect of carboplatin on TSA cells, while another showed that it can induce HMGB1 release and CRT exposure in murine CT26 and MC38 colon cancer cells [78,86].

Thus, contrarily to carboplatin and cisplatin, oxaliplatin might be able to induce an antitumor immune response through its capacity to induce ER stress, CRT exposure, and ATP and HMGB1 release in dying cancer cells. This was confirmed by “vaccination” experiments [35].

Many discrepancies are observed in the ability of some platinum derivatives to induce CRT exposure, ATP and/or HMGB1 release. This can be due to concentrations and time exposure differences, but also to the cell types used. Cell mutational status [87], lipid metabolism [88] or miRNA expression [89] can regulate ER stress and ICD induction by chemotherapy.

### 4.2. Strategies to Induce or Improve Platinum Derivative-Mediated ICD

One possible method to drive ICD induction by chemotherapy is to combine chemotherapeutic agents with other molecules able to trigger ICD features, such as CRT exposure, ATP or HMGB1 release. An automated epifluorescence microscopy-based platform to detect known biochemical hallmarks of ICD in human cancer cells enabled Kroemer’s team to find that cardiac glycosides, such as digoxin and digitoxin, both used in clinical practice, induced ICD [27]. Another compound identified is crizotinib. Although this tyrosine kinase inhibitor is able by itself to induce CRT exposure, ATP and HMGB1 release by NSCLCs, a vaccination effect of this compound requires cisplatin (or mitomycin C) treatment. It is worth noting that the combination of crizotinib and cisplatin together with anti-PD-1 and anti-CTLA4 immunotherapy has an important vaccine and antitumor effect [90]. Targeting another kinase, the serine/threonine kinase ataxia-telangiectasia mutated and RAD3-related (ATR), with its inhibitor VE-822, induces or increases ICD surrogate markers in the colon cancer model MC38 [91].

One way to induce CRT exposure at the cell surface is to induce eIF2α phosphorylation and ER stress (in association with chemotherapy). ER stress results from the accumulation of misfolded proteins, deregulation of redox and calcium homeostasis, or from protein glycosylation inhibition [92]. ER stress inducers, such as thapsigargin and tunicamycin, can restore the immunogenicity of cisplatin [81] in vitro and in vivo.

Some molecules found in nutrients or used as nutritional supplements have shown an effect on ICD. The vitamin B6 precursor pyridoxine induces or increases CRT exposure, and ATP and HMGB1 release from LLC cells [82]. Moreover, zinc dichloride entails eIF2α phosphorylation and CRT exposure on glioblastoma p53-deficient cells [93].

**Figure 3:**
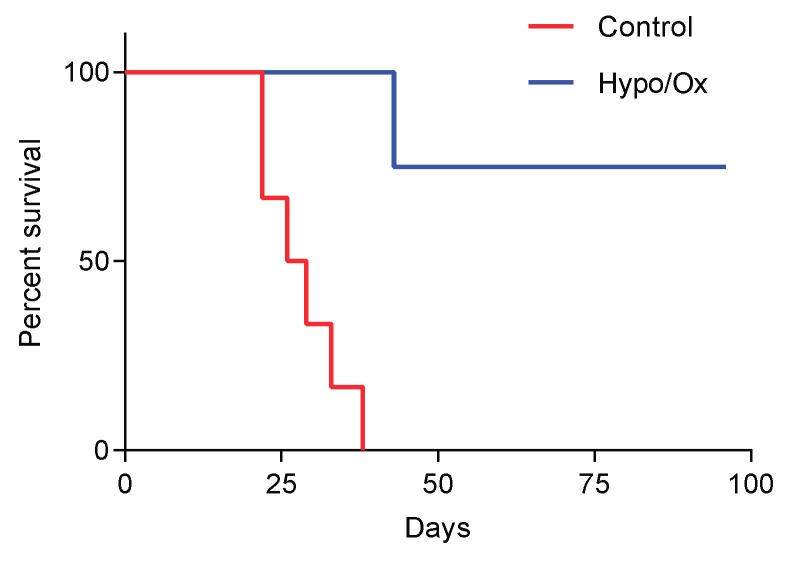
Oxaliplatin in hypotonic conditions treatment of peritoneal carcinomatosis vaccinates mice. Balb/c mice were intraperitoneally injected with 25,000 CT26 cells in 2 mL glucose 2.5% + 150 mg/L oxaliplatin (Hypo/ox). When animals were cured, 25,000 CT26 cells were s.c. injected, and tumor appearance was monitored. As a control, 25,000 CT26 cells were s.c. injected in Balb/c mice. Survival (n = 6 animals for Control group and n = 5 animals for Hypo/ox group) was represented using the Kaplan–Meier method. *p* < 0.01, using log-rank test. For more information on material and methods, see [25].

Molecules that have the ability to enhance platinum incorporation can also increase ICD. CBP501 (calmodulin binding peptide), an enhancer of platin uptake [94], increases cisplatin-mediated CRT exposure and HMGB1 release by CT26 cells and the vaccination potential of in vitro cisplatin -treated cells [95]. We also showed that hypotonic stress (induced by glucose 2.5%) can enhance platin incorporation by colon cancer cells, mainly through CTR1 oligomerization, which increases oxaliplatin, cisplatin and carboplatin cytoxicity. Furthermore, hypotonic conditions also allow oxaliplatin and cisplatin (but not carboplatin) to induce ICD. In a murine peritoneal carcinomatosis model (injection of CT26 cells in the peritoneal cavity), oxaliplatin in hypotonic conditions decreased the appearance of tumor nodules and increased mice survival. An increased CD8 T-cell recruitment and activation was observed at the tumor site. Moreover, treatment had no effect in immunodeficient mice or in CD8 T-cells depleted mice [25]. An s.c. injection of living CT26 cells, in peritoneal carcinomatosis mice cured by oxaliplatin in hypotonic conditions, does not induce tumor formation when compared with untreated mice that received CT26 cells injection, suggesting that this treatment induces mice vaccination (Figure 3).

Finally, in some mesothelioma cell lines, ONCOS-102, an adenovirus that specifically targets cancer cells, is able to increase cisplatin-induced CRT exposure, ATP and HMGB1 release [96].

## 5. Effects of Platinum Derivatives on Immune Cells

Another possibility for chemotherapy to affect antitumor immune response is to induce immune cell death or activation [97]. Cisplatin decreases the number of MDSCs in tumors of B16-bearing mice and in the spleen of CT26-bearing mice, with no effect on T (CD4 and CD8) and B cells [98,99]. Moreover, cisplatin also depleted PMN-MDSCs in a murine bladder cancer model, which was correlated with increased CD8^+^ T-cell number and activity [100]. We have shown that FOLFOX (5-FU/oxaliplatin/folinic acid) can decrease PNN-MDSCs number in patients’ blood [101]. However, we cannot discriminate the effects of oxaliplatin from those of 5-FU. In mice, oxaliplatin cannot decrease the number of MDSCs in CT26 tumors [26]. Finally, in vitro oxaliplatin decreases the number of MDSCs and their immunosuppressive functions [102].

In CT26 tumor-bearing mice, oxaliplatin can decrease both TAM1 and TAM2, but with the capacity to increase TAM1/TAM2 ratio [26]. This was correlated with in vitro experiments that show that oxaliplatin can inhibit the capacity of glioblastoma-conditioned media to induce M2 differentiation of murine macrophages, mainly by decreasing arginase-1 production [79]. Many studies have shown that activated macrophages can lead to tumor cell lysis. Macrophages pre-treated with cisplatin become active, as shown by their capacity to produce NO and pro-inflammatory cytokines and to express TLRs [103]. A pro-inflammatory effect of cisplatin was also reported in vitro with an additional cytotoxic effect of this compound on macrophages [104]. Further studies are required to precisely identify these effects on TAM1 and TAM2. Indirectly, cisplatin and carboplatin were shown to favor IL-6 and prostaglandin E2 production by cancer cells, allowing macrophage differentiation into M2 subtype [105].

Oxaliplatin may indirectly impact DCs due to its ability to induce ICD [83]. Moreover, cisplatin only affects pSTAT6 in human DCs, but no effect on cell function was observed [106,107].

In a murine lung cancer model, cisplatin increased intra-tumoral APCs and the expression of co-stimulatory molecules, then leading to IFNγ and TNFα production by CD8^+^ T cells [108]. We also found similar effects of oxaliplatin on CD8^+^ T cells in the murine CT26 tumor model [26]. In ovarian cancer patients, carboplatin does not affect CD8^+^ T-cell proportion in blood, but it increases their capacity to produce IFNγ [109]. In NSCLC patients treated with paclitaxel/carboplatin/bevacizumab, CD8^+^ T-cell proliferation in peripheral blood is slightly increased [110].

Discrepancies between observed effects of platinum derivatives on immune cells can be explained by the model (human, mouse in vitro, in vivo) and chemotherapy regimen (time, concentrations) used. Further studies should be performed to further clarify these discrepancies.

## 6. Platinum Derivatives and Immune Checkpoints

Platinum derivatives and anti-PD-1/PD-L1 use in cancer patients’ treatment are linked. In some studies, anti-PD-1/PD-L1 were used in platinum derivative-resistant cancers and in others, platinum derivatives were shown to increase anti-PD-1/PD-L1 efficiency (Table 2). In clinical trials, patients are treated with several chemotherapeutic compounds with or without radiotherapy, making it difficult to identify individual effects of one particular compound. However, platinum derivative-based treatment in association with anti-PD-1 or anti-PD-L1 has been tested in many clinical trials with heterogeneous effects [111,112,113,114]. Many pre-clinical studies in mice have shown the limitations of anti-PD-1/PD-L1 therapy. This raises the question of how immunotherapy efficacy should be evaluated. Are there only a few patients eligible for these treatments, and does chemotherapy increase or inhibit immunotherapy? Is there a cancer type specificity?

In a murine colon cancer model, oxaliplatin had no impact on PD-1 expression on MDSCs, TAMs and CT26 tumor cells in vivo. However, it induced PD-1 expression on CD8^+^ T cells, which was associated with a decreased proliferation. The association of oxaliplatin with trifluridine/tipiracil favors PD-L1 expression on tumor cells and improves anti-PD-1 therapy [26,115]. FOLFOX and, to a lesser extent, oxaliplatin induce PD-L1 expression at CT26 tumor cells’ surface in vivo. Although the real effect of oxaliplatin is not known, FOLFOX also enhances PD-L1 expression in tumor cells from patients [115].

The TONIC trial on metastatic triple-negative breast cancer patients shows that doxorubicin or cisplatin allows tumors to have the capacity to respond to anti-PD-1. This was deduced from high response rates to anti-PD-1 and from upregulation of immune-related gene sets [116]. Cisplatin increased radiotherapy + immunotherapy (anti-PD-1 + anti-CD137) effects on tumor growth in a murine AT-3 breast cancer model [117].

In human head and neck squamous cell carcinoma (HNSCC) cell lines, cisplatin could not increase PD-L1 expression in any of the cell lines tested. This observation was confirmed in patients by comparing the expression before and after treatment, using IHC [118]. In a syngeneic mouse model of HNSCC, cisplatin impaired T-cell function. Its association with anti-PD-L1/PD-1 was shown to slow down tumor growth and to improve survival without any significant effect on cisplatin-induced toxicities, or on the number and the function of tumor-infiltrating immune cells [119]. Similar results were observed in another study. Cisplatin and oxaliplatin induce PD-L1 expression in some but not all murine HNSCC cells, and the therapeutic association of cisplatin or oxaliplatin with anti-PD-1 is as effective as monotherapy [73].

Similar observations were shown in lung cancer patients, with different histological characteristics and treated with cisplatin or carboplatin-derived chemotherapy [120]. In NSCLC patients treated with paclitaxel/carboplatin/bevacizumab, proliferating peripheral blood CD8^+^ T cells express a higher level of PD-1 and CTLA-4 compared to non-proliferating CD8^+^ T cells [110]. In vitro, small cell lung cancer (SCLC) cells resistant to cisplatin after continuous exposure to low doses express higher levels of PD-1 and PD-L1, leading to survival and proliferation [121]. In lung cancer models, cisplatin was described to increase PD-L1 expression in patient biopsies, in tumor cells in vitro, and in murine tumor models. This increase in PD-L1 expression was also correlated with a more important antitumor effect of cisplatin combined with anti-PD-L1 in a murine lung carcinoma model [122].

Similar results were obtained in murine ovary tumor models [123]. Moreover, nivolumab inhibits platinum-resistant ovarian cancer cells by inducing cell apoptosis in vitro [124].

In vitro treatment of mesothelioma cell lines or PBMCs with oxaliplatin or cisplatin (IC20) has no impact on the expression of PD-1, PD-L1/2, TIM-3 and other immune checkpoints [125].

In contrast, cisplatin seems to be able to increase PD-L1 expression on H22 hepatoma cells both in vitro and in vivo [126].

In bladder cancer cell lines, cisplatin is able to induce the expression of PD-L1 in vitro [127]. The combination of cisplatin and anti-PD-L1 is also synergic in murine thymoma. Yet, it seems to be correlated with PD-1 expression on CD8^+^ T cells [128].

Even if platinum derivatives seem to induce PD-1/PD-L1 expression, some discrepancies were observed. This might be explained by the cell lines (more about the genetic background than tumor type) and compound concentrations used, or by a possible indirect effect of platinum derivatives on cancer cells or neighboring cells that will in turn regulate PD-L1 expression. To our knowledge, no effect of platinum derivatives on other immune checkpoints were reported.

## 7. Conclusions

Cisplatin, carboplatin and oxaliplatin play an important role in antitumor immune response. Oxaliplatin seems to be the compound with the strongest immunogenicity, mainly by its capacity to induce ICD. However, cisplatin and carboplatin also impact this immune response, and many studies showed that some molecules and/or conditions of treatment may help these platinum derivatives, particularly cisplatin, to induce ICD. Further studies are required to understand the mechanisms responsible for the capacity of platinum derivatives to improve antitumor immune response and to favor immune checkpoint inhibitors’ efficacy, such as PD-1/PD-L1 and other immune checkpoints.

## Figures and Tables

**Table 1:** Main characteristics of platinum derivatives.

	Cisplatin	Carboplatin	Oxaliplatin
**Generation (FDA Agreement Date)**	First (1978)	Second (1989)	Third (2002)
**Structure**	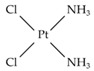	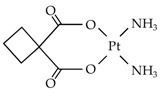	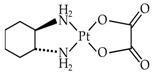
**Main Cellular Transporters**	CTR1 [58] OCT1, OCT2 [59] ATP7A and ATP7B [60] Na^+^, K^+^ -ATPase pump [60] VRAC [60]	CTR1 [58] ATP7B [60]	Passive absorption [61] CTR1 [58,60] OCT1, OCT2 [60] Na^+^, K^+^ -ATPase pump [60] MATEs [62]
**DNA Adducts**	Intra-strand and less frequently inter-strand connections [60]	Fewer intra-strand and less frequently inter-strand connections than with cisplatin at equimolar concentrations [60,63]	Intra and inter-strand connections more stable and inducing a more important DNA distortion [60]
**Repair Mechanisms**	NER and MMR [64,65]	NER and MMR [65]	NER [65]
**Type of Cancer**	Ovary, testis, bladder, colon, rectum, lung or head and neck cancers [66]	Ovary, lung and ENT Reduced efficacy in testis, bladder and epidermoid head and neck cancers [67]	Stage II/III colon cancers, metastatic colorectal cancers and NSCLCs [68]
**Side Effects**	Nausea, vomiting, nephrotoxicity, myelosuppression (thrombocytopenia, leucopenia, anemia) and peripheral sensory neuropathy (ototoxicity) [64]	Less important neurotoxicity and ototoxicity than cisplatin Serious myelosuppression strong thrombocytopenia, neutropenia and anemia [60,66]	Sensorial neuropathy but no hepatic or kidney toxicity [69]

**Table 2:** Immune checkpoints and platinum derivatives crosstalk.

		Cisplatin	Carboplatin	Oxaliplatin
**Mouse**	**CT26 Colon Cancer**			↗ PD-1 on CD8^+^ T-cells [26,115] Oxaliplatin with trifluridine/tipiracil or FOLFOX ↗ PD-1 on tumor cells and ↗ anti-PD-1 therapeutic effect [26,115]
	**AT-3 Breast Cancer**	↗ radiotherapy + immunotherapy (anti-PD-1 + anti-CD137) effects [117]		
	**HNSCC**	Impaired T-cell function [119] ↗ PD-L1 expression on cancer cells [73] Association with anti-PD-L1/PD-1 improve therapeutic effects [119] or not [73]		↗ PD-L1 expression on cancer cells [73] Association with anti-PD-L1/PD-1 has no effect [73]
	**NSCLC**	↗ PD-L1 expression in tumors [122] ↗ cisplatin combined with anti-PD-L1 antitumor effect [122]		
	**Thymoma**	Cisplatin and anti-PD-L1 ↗ therapeutic effect [128]		
	**H22 Hepatoma**	↗ PD-L1 expression on tumor cells [126]		
**Human**	**Metastatic Triple-Negative Breast Cancer**	↗ anti-PD-1 patient response [116]		
	**HNSCC**	No effect on PD-L1 expression [118]		
	**NSCLC**	↗ PD-L1 expression in patient biopsies [122]	Paclitaxel/carboplatin/bevacizumab ↗ PD-1 and CTLA-4 on proliferating peripheral CD8^+^ T-cells [110]	

## References

[1] Hanahan D., Weinberg R.A. (2011). Hallmarks of cancer: The next generation. Cell.

[2] McWhinney S.R., Goldberg R.M., McLeod H.L. (2009). Platinum neurotoxicity pharmacogenetics. Mol. Cancer Ther..

[3] Kim S.Y., Song X., Zhang L., Bartlett D.L., Lee Y.J. (2014). Role of Bcl-xL/Beclin-1 in interplay between apoptosis and autophagy in oxaliplatin and bortezomib-induced cell death. Biochem. Pharmacol..

[4] Galluzzi L., Vitale I., Aaronson S.A., Abrams J.M., Adam D., Agostinis P., Alnemri E.S., Altucci L., Amelio I., Andrews D.W. (2018). Molecular mechanisms of cell death: Recommendations of the Nomenclature Committee on Cell Death 2018. Cell Death Differ..

[5] Casares N., Pequignot M.O., Tesniere A., Ghiringhelli F., Roux S., Chaput N., Schmitt E., Hamai A., Hervas-Stubbs S., Obeid M. (2005). Caspase-dependent immunogenicity of doxorubicin-induced tumor cell death. J. Exp. Med..

[6] Panaretakis T., Kepp O., Brockmeier U., Tesniere A., Bjorklund A.C., Chapman D.C., Durchschlag M., Joza N., Pierron G., van Endert P. (2009). Mechanisms of pre-apoptotic calreticulin exposure in immunogenic cell death. EMBO J..

[7] Galluzzi L., Buque A., Kepp O., Zitvogel L., Kroemer G. (2017). Immunogenic cell death in cancer and infectious disease. Nat. Rev. Immunol..

[8] Bezu L., Gomes-de-Silva L.C., Dewitte H., Breckpot K., Fucikova J., Spisek R., Galluzzi L., Kepp O., Kroemer G. (2015). Combinatorial strategies for the induction of immunogenic cell death. Front. Immunol..

[9] Obeid M., Tesniere A., Ghiringhelli F., Fimia G.M., Apetoh L., Perfettini J.L., Castedo M., Mignot G., Panaretakis T., Casares N. (2007). Calreticulin exposure dictates the immunogenicity of cancer cell death. Nat. Med..

[10] Garg A.D., Krysko D.V., Verfaillie T., Kaczmarek A., Ferreira G.B., Marysael T., Rubio N., Firczuk M., Mathieu C., Roebroek A.J. (2012). A novel pathway combining calreticulin exposure and ATP secretion in immunogenic cancer cell death. EMBO J..

[11] Montico B., Nigro A., Casolaro V., Dal Col J. (2018). Immunogenic Apoptosis as a Novel Tool for Anticancer Vaccine Development. Int. J. Mol. Sci..

[12] Martins I., Wang Y., Michaud M., Ma Y., Sukkurwala A.Q., Shen S., Kepp O., Metivier D., Galluzzi L., Perfettini J.L. (2014). Molecular mechanisms of ATP secretion during immunogenic cell death. Cell Death Differ..

[13] Derangere V., Chevriaux A., Courtaut F., Bruchard M., Berger H., Chalmin F., Causse S.Z., Limagne E., Vegran F., Ladoire S. (2014). Liver X receptor beta activation induces pyroptosis of human and murine colon cancer cells. Cell Death Differ..

[14] Ghiringhelli F., Apetoh L., Tesniere A., Aymeric L., Ma Y., Ortiz C., Vermaelen K., Panaretakis T., Mignot G., Ullrich E. (2009). Activation of the NLRP3 inflammasome in dendritic cells induces IL-1beta-dependent adaptive immunity against tumors. Nat. Med..

[15] Senovilla L., Aranda F., Galluzzi L., Kroemer G. (2014). Impact of myeloid cells on the efficacy of anticancer chemotherapy. Curr. Opin. Immunol..

[16] Ghiringhelli F., Bruchard M., Chalmin F., Rebe C. (2012). Production of adenosine by ectonucleotidases: A key factor in tumor immunoescape. J. Biomed Biotechnol..

[17] Luo Y., Chihara Y., Fujimoto K., Sasahira T., Kuwada M., Fujiwara R., Fujii K., Ohmori H., Kuniyasu H. (2013). High mobility group box 1 released from necrotic cells enhances regrowth and metastasis of cancer cells that have survived chemotherapy. Eur. J. Cancer.

[18] Bonaldi T., Talamo F., Scaffidi P., Ferrera D., Porto A., Bachi A., Rubartelli A., Agresti A., Bianchi M.E. (2003). Monocytic cells hyperacetylate chromatin protein HMGB1 to redirect it towards secretion. EMBO J..

[19] Dumitriu I.E., Bianchi M.E., Bacci M., Manfredi A.A., Rovere-Querini P. (2007). The secretion of HMGB1 is required for the migration of maturing dendritic cells. J. Leukoc. Biol..

[20] Semino C., Angelini G., Poggi A., Rubartelli A. (2005). NK/iDC interaction results in IL-18 secretion by DCs at the synaptic cleft followed by NK cell activation and release of the DC maturation factor HMGB1. Blood.

[21] Lotze M.T., Tracey K.J. (2005). High-mobility group box 1 protein (HMGB1): Nuclear weapon in the immune arsenal. Nat. Rev. Immunol..

[22] Scaffidi P., Misteli T., Bianchi M.E. (2002). Release of chromatin protein HMGB1 by necrotic cells triggers inflammation. Nature.

[23] Schiraldi M., Raucci A., Munoz L.M., Livoti E., Celona B., Venereau E., Apuzzo T., De Marchis F., Pedotti M., Bachi A. (2012). HMGB1 promotes recruitment of inflammatory cells to damaged tissues by forming a complex with CXCL12 and signaling via CXCR4. J. Exp. Med..

[24] Sistigu A., Yamazaki T., Vacchelli E., Chaba K., Enot D.P., Adam J., Vitale I., Goubar A., Baracco E.E., Remedios C. (2014). Cancer cell-autonomous contribution of type I interferon signaling to the efficacy of chemotherapy. Nat. Med..

[25] Demontoux L., Derangere V., Pilot T., Thinselin C., Chevriaux A., Chalmin F., Bouyer F., Ghiringhelli F., Rebe C. (2019). Hypotonic stress enhances colon cancer cell death induced by platinum derivatives and immunologically improves antitumor efficacy of intraperitoneal chemotherapy. Int. J. Cancer.

[26] Limagne E., Thibaudin M., Nuttin L., Spill A., Derangere V., Fumet J.D., Amellal N., Peranzoni E., Cattan V., Ghiringhelli F. (2019). Trifluridine/tipiracil plus oxaliplatin improves PD-1 blockade in colorectal cancer by inducing immunogenic cell death and depleting macrophages. Cancer Immunol. Res..

[27] Menger L., Vacchelli E., Adjemian S., Martins I., Ma Y., Shen S., Yamazaki T., Sukkurwala A.Q., Michaud M., Mignot G. (2012). Cardiac glycosides exert anticancer effects by inducing immunogenic cell death. Sci. Transl. Med..

[28] Sukkurwala A.Q., Adjemian S., Senovilla L., Michaud M., Spaggiari S., Vacchelli E., Baracco E.E., Galluzzi L., Zitvogel L., Kepp O. (2014). Screening of novel immunogenic cell death inducers within the NCI Mechanistic Diversity Set. Oncoimmunology.

[29] Van Schadewijk A., van’t Wout E.F., Stolk J., Hiemstra P.S. (2012). A quantitative method for detection of spliced X-box binding protein-1 (XBP1) mRNA as a measure of endoplasmic reticulum (ER) stress. Cell Stress Chaperones.

[30] Senovilla L., Vitale I., Martins I., Tailler M., Pailleret C., Michaud M., Galluzzi L., Adjemian S., Kepp O., Niso-Santano M. (2012). An immunosurveillance mechanism controls cancer cell ploidy. Science.

[31] Martins I., Tesniere A., Kepp O., Michaud M., Schlemmer F., Senovilla L., Seror C., Metivier D., Perfettini J.L., Zitvogel L. (2009). Chemotherapy induces ATP release from tumor cells. Cell Cycle.

[32] Sorensen C.E., Novak I. (2001). Visualization of ATP release in pancreatic acini in response to cholinergic stimulus. Use of fluorescent probes and confocal microscopy. J. Biol. Chem..

[33] Tesniere A., Schlemmer F., Boige V., Kepp O., Martins I., Ghiringhelli F., Aymeric L., Michaud M., Apetoh L., Barault L. (2010). Immunogenic death of colon cancer cells treated with oxaliplatin. Oncogene.

[34] Zhou H., Forveille S., Sauvat A., Yamazaki T., Senovilla L., Ma Y., Liu P., Yang H., Bezu L., Muller K. (2016). The oncolytic peptide LTX-315 triggers immunogenic cell death. Cell Death Dis..

[35] Kepp O., Senovilla L., Vitale I., Vacchelli E., Adjemian S., Agostinis P., Apetoh L., Aranda F., Barnaba V., Bloy N. (2014). Consensus guidelines for the detection of immunogenic cell death. Oncoimmunology.

[36] Marigo I., Dolcetti L., Serafini P., Zanovello P., Bronte V. (2008). Tumor-induced tolerance and immune suppression by myeloid derived suppressor cells. Immunol. Rev..

[37] Crowley M., Inaba K., Steinman R.M. (1990). Dendritic cells are the principal cells in mouse spleen bearing immunogenic fragments of foreign proteins. J. Exp. Med..

[38] Steinman R.M. (1991). The dendritic cell system and its role in immunogenicity. Annu. Rev. Immunol..

[39] Mills C.D., Kincaid K., Alt J.M., Heilman M.J., Hill A.M. (2000). M-1/M-2 macrophages and the Th1/Th2 paradigm. J. Immunol..

[40] Martinez F.O., Sica A., Mantovani A., Locati M. (2008). Macrophage activation and polarization. Front. Biosci..

[41] Gabrilovich D.I., Bronte V., Chen S.H., Colombo M.P., Ochoa A., Ostrand-Rosenberg S., Schreiber H. (2007). The terminology issue for myeloid-derived suppressor cells. Cancer Res..

[42] Almand B., Clark J.I., Nikitina E., van Beynen J., English N.R., Knight S.C., Carbone D.P., Gabrilovich D.I. (2001). Increased production of immature myeloid cells in cancer patients: A mechanism of immunosuppression in cancer. J. Immunol..

[43] Diaz-Montero C.M., Salem M.L., Nishimura M.I., Garrett-Mayer E., Cole D.J., Montero A.J. (2009). Increased circulating myeloid-derived suppressor cells correlate with clinical cancer stage, metastatic tumor burden, and doxorubicin-cyclophosphamide chemotherapy. Cancer Immunol. Immunother..

[44] Nagaraj S., Gupta K., Pisarev V., Kinarsky L., Sherman S., Kang L., Herber D.L., Schneck J., Gabrilovich D.I. (2007). Altered recognition of antigen is a mechanism of CD8+ T cell tolerance in cancer. Nat. Med..

[45] Tcyganov E., Mastio J., Chen E., Gabrilovich D.I. (2018). Plasticity of myeloid-derived suppressor cells in cancer. Curr. Opin. Immunol..

[46] Shiku H. (2003). Importance of CD4+ helper T-cells in antitumor immunity. Int. J. Hematol..

[47] Marrogi A.J., Munshi A., Merogi A.J., Ohadike Y., El-Habashi A., Marrogi O.L., Freeman S.M. (1997). Study of tumor infiltrating lymphocytes and transforming growth factor-beta as prognostic factors in breast carcinoma. Int. J. Cancer..

[48] Galon J., Costes A., Sanchez-Cabo F., Kirilovsky A., Mlecnik B., Lagorce-Pages C., Tosolini M., Camus M., Berger A., Wind P. (2006). Type, density, and location of immune cells within human colorectal tumors predict clinical outcome. Science.

[49] Hiraoka K., Miyamoto M., Cho Y., Suzuoki M., Oshikiri T., Nakakubo Y., Itoh T., Ohbuchi T., Kondo S., Katoh H. (2006). Concurrent infiltration by CD8+ T cells and CD4+ T cells is a favourable prognostic factor in non-small-cell lung carcinoma. Br. J. Cancer.

[50] Knutson K.L., Disis M.L. (2005). Tumor antigen-specific T helper cells in cancer immunity and immunotherapy. Cancer Immunol. Immunother..

[51] Durgeau A., Virk Y., Corgnac S., Mami-Chouaib F. (2018). Recent Advances in Targeting CD8 T-Cell Immunity for More Effective Cancer Immunotherapy. Front. Immunol..

[52] Kyi C., Postow M.A. (2016). Immune checkpoint inhibitor combinations in solid tumors: Opportunities and challenges. Immunotherapy.

[53] Pedoeem A., Azoulay-Alfaguter I., Strazza M., Silverman G.J., Mor A. (2014). Programmed death-1 pathway in cancer and autoimmunity. Clin. Immunol..

[54] Postow M.A., Callahan M.K., Wolchok J.D. (2015). Immune Checkpoint Blockade in Cancer Therapy. J. Clin. Oncol. Off. J. Am. Soc. Clin. Oncol..

[55] Butte M.J., Keir M.E., Phamduy T.B., Sharpe A.H., Freeman G.J. (2007). Programmed death-1 ligand 1 interacts specifically with the B7-1 costimulatory molecule to inhibit T cell responses. Immunity.

[56] Schneider H., Rudd C.E. (2014). Diverse mechanisms regulate the surface expression of immunotherapeutic target ctla-4. Front. Immunol..

[57] Giuroiu I., Weber J. (2017). Novel Checkpoints and Cosignaling Molecules in Cancer Immunotherapy. Cancer J..

[58] Howell S.B., Safaei R., Larson C.A., Sailor M.J. (2010). Copper transporters and the cellular pharmacology of the platinum-containing cancer drugs. Mol. Pharm..

[59] Ciarimboli G., Ludwig T., Lang D., Pavenstadt H., Koepsell H., Piechota H.J., Haier J., Jaehde U., Zisowsky J., Schlatter E. (2005). Cisplatin nephrotoxicity is critically mediated via the human organic cation transporter 2. Am. J. Pathol..

[60] Dilruba S., Kalayda G.V. (2016). Platinum-based drugs: Past, present and future. Cancer Chemother. Pharm..

[61] Ndagi U., Mhlongo N., Soliman M.E. (2017). Metal complexes in cancer therapy—an update from drug design perspective. Drug Des. Dev. Ther..

[62] Yonezawa A., Inui K. (2011). Organic cation transporter OCT/SLC22A and H(+)/organic cation antiporter MATE/SLC47A are key molecules for nephrotoxicity of platinum agents. Biochem. Pharm..

[63] Kelland L. (2007). The resurgence of platinum-based cancer chemotherapy. Nat. Rev. Cancer.

[64] Rancoule C., Guy J.B., Vallard A., Ben Mrad M., Rehailia A., Magne N. (2017). 50th anniversary of cisplatin. Bull. Cancer.

[65] van Zyl B., Tang D., Bowden N.A. (2018). Biomarkers of platinum resistance in ovarian cancer: What can we use to improve treatment. Endocr. Relat. Cancer.

[66] Dasari S., Tchounwou P.B. (2014). Cisplatin in cancer therapy: Molecular mechanisms of action. Eur. J. Pharm..

[67] Stewart D.J. (2007). Mechanisms of resistance to cisplatin and carboplatin. Crit. Rev. Oncol. Hematol..

[68] Falzone L., Salomone S., Libra M. (2018). Evolution of Cancer Pharmacological Treatments at the Turn of the Third Millennium. Front. Pharm..

[69] Elias D., Raynard B., Bonnay M., Pocard M. (2006). Heated intra-operative intraperitoneal oxaliplatin alone and in combination with intraperitoneal irinotecan: Pharmacologic studies. Eur. J. Surg. Oncol..

[70] Wheate N.J., Walker S., Craig G.E., Oun R. (2010). The status of platinum anticancer drugs in the clinic and in clinical trials. Dalton. Trans..

[71] de Biasi A.R., Villena-Vargas J., Adusumilli P.S. (2014). Cisplatin-induced antitumor immunomodulation: A review of preclinical and clinical evidence. Clin. Cancer Res..

[72] Wan S., Pestka S., Jubin R.G., Lyu Y.L., Tsai Y.C., Liu L.F. (2012). Chemotherapeutics and radiation stimulate MHC class I expression through elevated interferon-beta signaling in breast cancer cells. PLoS ONE.

[73] Park S.J., Ye W., Xiao R., Silvin C., Padget M., Hodge J.W., Van Waes C., Schmitt N.C. (2019). Cisplatin and oxaliplatin induce similar immunogenic changes in preclinical models of head and neck cancer. Oral. Oncol..

[74] Vacchelli E., Aranda F., Eggermont A., Galon J., Sautes-Fridman C., Cremer I., Zitvogel L., Kroemer G., Galluzzi L. (2014). Trial Watch: Chemotherapy with immunogenic cell death inducers. Oncoimmunology.

[75] Michaud M., Martins I., Sukkurwala A.Q., Adjemian S., Ma Y., Pellegatti P., Shen S., Kepp O., Scoazec M., Mignot G. (2011). Autophagy-dependent anticancer immune responses induced by chemotherapeutic agents in mice. Science.

[76] Zhao X., Yang K., Zhao R., Ji T., Wang X., Yang X., Zhang Y., Cheng K., Liu S., Hao J. (2016). Inducing enhanced immunogenic cell death with nanocarrier-based drug delivery systems for pancreatic cancer therapy. Biomaterials.

[77] Sun F., Cui L., Li T., Chen S., Song J., Li D. (2019). Oxaliplatin induces immunogenic cells death and enhances therapeutic efficacy of checkpoint inhibitor in a model of murine lung carcinoma. J. Recept. Signal Transduct. Res..

[78] Golden E.B., Frances D., Pellicciotta I., Demaria S., Helen Barcellos-Hoff M., Formenti S.C. (2014). Radiation fosters dose-dependent and chemotherapy-induced immunogenic cell death. Oncoimmunology.

[79] Roberts N.B., Alqazzaz A., Hwang J.R., Qi X., Keegan A.D., Kim A.J., Winkles J.A., Woodworth G.F. (2018). Oxaliplatin disrupts pathological features of glioma cells and associated macrophages independent of apoptosis induction. J. Neurooncol..

[80] Sagwal S.K., Pasqual-Melo G., Bodnar Y., Gandhirajan R.K., Bekeschus S. (2018). Combination of chemotherapy and physical plasma elicits melanoma cell death via upregulation of SLC22A16. Cell Death Dis..

[81] Martins I., Kepp O., Schlemmer F., Adjemian S., Tailler M., Shen S., Michaud M., Menger L., Gdoura A., Tajeddine N. (2011). Restoration of the immunogenicity of cisplatin-induced cancer cell death by endoplasmic reticulum stress. Oncogene.

[82] Aranda F., Bloy N., Pesquet J., Petit B., Chaba K., Sauvat A., Kepp O., Khadra N., Enot D., Pfirschke C. (2015). Immune-dependent antineoplastic effects of cisplatin plus pyridoxine in non-small-cell lung cancer. Oncogene.

[83] Di Blasio S., Wortel I.M., van Bladel D.A., de Vries L.E., Duiveman-de Boer T., Worah K., de Haas N., Buschow S.I., de Vries I.J., Figdor C.G. (2016). Human CD1c(+) DCs are critical cellular mediators of immune responses induced by immunogenic cell death. Oncoimmunology.

[84] Luo R., Firat E., Gaedicke S., Guffart E., Watanabe T., Niedermann G. (2019). Cisplatin facilitates radiation-induced abscopal effects in conjunction with PD-1 checkpoint blockade through CXCR3/CXCL10-mediated T cell recruitment. Clin. Cancer Res..

[85] Li G., Tian L., Hou J.M., Ding Z.Y., He Q.M., Feng P., Wen Y.J., Xiao F., Yao B., Zhang R. (2005). Improved therapeutic effectiveness by combining recombinant CXC chemokine ligand 10 with Cisplatin in solid tumors. Clin. Cancer Res..

[86] Schaer D.A., Geeganage S., Amaladas N., Lu Z.H., Rasmussen E.R., Sonyi A., Chin D., Capen A., Li Y., Meyer C.M. (2019). The folate pathway inhibitor pemetrexed pleiotropically enhances effects of cancer immunotherapy. Clin. Cancer Res..

[87] Cubillos-Ruiz J.R., Bettigole S.E., Glimcher L.H. (2017). Tumorigenic and Immunosuppressive Effects of Endoplasmic Reticulum Stress in Cancer. Cell.

[88] Cotte A.K., Aires V., Fredon M., Limagne E., Derangere V., Thibaudin M., Humblin E., Scagliarini A., de Barros J.P., Hillon P. (2018). Lysophosphatidylcholine acyltransferase 2-mediated lipid droplet production supports colorectal cancer chemoresistance. Nat. Commun..

[89] Colangelo T., Polcaro G., Ziccardi P., Muccillo L., Galgani M., Pucci B., Milone M.R., Budillon A., Santopaolo M., Mazzoccoli G. (2016). The miR-27a-calreticulin axis affects drug-induced immunogenic cell death in human colorectal cancer cells. Cell Death Dis..

[90] Liu P., Zhao L., Pol J., Levesque S., Petrazzuolo A., Pfirschke C., Engblom C., Rickelt S., Yamazaki T., Iribarren K. (2019). Crizotinib-induced immunogenic cell death in non-small cell lung cancer. Nat. Commun..

[91] Combes E., Andrade A.F., Tosi D., Michaud H.A., Coquel F., Garambois V., Desigaud D., Jarlier M., Coquelle A., Pasero P. (2019). Inhibition of Ataxia-Telangiectasia Mutated and RAD3-Related (ATR) Overcomes Oxaliplatin Resistance and Promotes Antitumor Immunity in Colorectal Cancer. Cancer Res..

[92] Kepp O., Semeraro M., Bravo-San Pedro J.M., Bloy N., Buque A., Huang X., Zhou H., Senovilla L., Kroemer G., Galluzzi L. (2015). eIF2alpha phosphorylation as a biomarker of immunogenic cell death. Semin. Cancer Biol..

[93] Cirone M., Garufi A., Di Renzo L., Granato M., Faggioni A., D’Orazi G. (2013). Zinc supplementation is required for the cytotoxic and immunogenic effects of chemotherapy in chemoresistant p53-functionally deficient cells. Oncoimmunology.

[94] Mine N., Yamamoto S., Saito N., Yamazaki S., Suda C., Ishigaki M., Kufe D.W., Von Hoff D.D., Kawabe T. (2011). CBP501-calmodulin binding contributes to sensitizing tumor cells to cisplatin and bleomycin. Mol. Cancer Ther..

[95] Sakakibara K., Sato T., Kufe D.W., VonHoff D.D., Kawabe T. (2017). CBP501 induces immunogenic tumor cell death and CD8 T cell infiltration into tumors in combination with platinum, and increases the efficacy of immune checkpoint inhibitors against tumors in mice. Oncotarget.

[96] Kuryk L., Haavisto E., Garofalo M., Capasso C., Hirvinen M., Pesonen S., Ranki T., Vassilev L., Cerullo V. (2016). Synergistic anti-tumor efficacy of immunogenic adenovirus ONCOS-102 (Ad5/3-D24-GM-CSF) and standard of care chemotherapy in preclinical mesothelioma model. Int. J. Cancer.

[97] Rebe C., Ghiringhelli F. (2015). Cytotoxic effects of chemotherapy on cancer and immune cells: How can it be modulated to generate novel therapeutic strategies?. Future. Oncol..

[98] Gobbo J., Marcion G., Cordonnier M., Dias A.M.M., Pernet N., Hammann A., Richaud S., Mjahed H., Isambert N., Clausse V. (2016). Restoring Anticancer Immune Response by Targeting Tumor-Derived Exosomes With a HSP70 Peptide Aptamer. J. Natl. Cancer Inst..

[99] Huang X., Cui S., Shu Y. (2016). Cisplatin selectively downregulated the frequency and immunoinhibitory function of myeloid-derived suppressor cells in a murine B16 melanoma model. Immunol. Res..

[100] Wu K., Tan M.Y., Jiang J.T., Mu X.Y., Wang J.R., Zhou W.J., Wang X., Li M.Q., He Y.Y., Liu Z.H. (2018). Cisplatin inhibits the progression of bladder cancer by selectively depleting G-MDSCs: A novel chemoimmunomodulating strategy. Clin. Immunol..

[101] Limagne E., Euvrard R., Thibaudin M., Rebe C., Derangere V., Chevriaux A., Boidot R., Vegran F., Bonnefoy N., Vincent J. (2016). Accumulation of MDSC and Th17 Cells in Patients with Metastatic Colorectal Cancer Predicts the Efficacy of a FOLFOX-Bevacizumab Drug Treatment Regimen. Cancer Res..

[102] Kim N.R., Kim Y.J. (2019). Oxaliplatin regulates myeloid-derived suppressor cell-mediated immunosuppression via downregulation of nuclear factor-kappaB signaling. Cancer Med..

[103] Chauhan P., Sodhi A., Shrivastava A. (2009). Cisplatin primes murine peritoneal macrophages for enhanced expression of nitric oxide, proinflammatory cytokines, TLRs, transcription factors and activation of MAP kinases upon co-incubation with L929 cells. Immunobiology.

[104] Mai F.Y., He P., Ye J.Z., Xu L.H., Ouyang D.Y., Li C.G., Zeng Q.Z., Zeng C.Y., Zhang C.C., He X.H. (2019). Caspase-3-mediated GSDME activation contributes to cisplatin- and doxorubicin-induced secondary necrosis in mouse macrophages. Cell Prolif..

[105] Dijkgraaf E.M., Heusinkveld M., Tummers B., Vogelpoel L.T., Goedemans R., Jha V., Nortier J.W., Welters M.J., Kroep J.R., van der Burg S.H. (2013). Chemotherapy alters monocyte differentiation to favor generation of cancer-supporting M2 macrophages in the tumor microenvironment. Cancer Res..

[106] de Haas N., de Koning C., di Blasio S., Florez-Grau G., de Vries I.J.M., Hato S.V. (2019). STAT Family Protein Expression and Phosphorylation State during moDC Development Is Altered by Platinum-Based Chemotherapeutics. J. Immunol. Res..

[107] Aymeric L., Apetoh L., Ghiringhelli F., Tesniere A., Martins I., Kroemer G., Smyth M.J., Zitvogel L. (2010). Tumor cell death and ATP release prime dendritic cells and efficient anticancer immunity. Cancer Res..

[108] Beyranvand Nejad E., van der Sluis T.C., van Duikeren S., Yagita H., Janssen G.M., van Veelen P.A., Melief C.J., van der Burg S.H., Arens R. (2016). Tumor Eradication by Cisplatin Is Sustained by CD80/86-Mediated Costimulation of CD8+ T Cells. Cancer Res..

[109] Wu X., Feng Q.M., Wang Y., Shi J., Ge H.L., Di W. (2010). The immunologic aspects in advanced ovarian cancer patients treated with paclitaxel and carboplatin chemotherapy. Cancer Immunol. Immunother..

[110] de Goeje P.L., Poncin M., Bezemer K., Kaijen-Lambers M.E.H., Groen H.J.M., Smit E.F., Dingemans A.C., Kunert A., Hendriks R.W., Aerts J. (2019). Induction of Peripheral Effector CD8 T-cell Proliferation by Combination of Paclitaxel, Carboplatin, and Bevacizumab in Non-small Cell Lung Cancer Patients. Clin. Cancer Res..

[111] Frederickson A.M., Arndorfer S., Zhang I., Lorenzi M., Insinga R., Arunachalam A., Burke T.A., Simon G.R. (2019). Pembrolizumab plus chemotherapy for first-line treatment of metastatic nonsquamous non-small-cell lung cancer: A network meta-analysis. Immunotherapy.

[112] Pacheco J.M., Camidge D.R., Doebele R.C., Schenk E. (2019). A Changing of the Guard: Immune Checkpoint Inhibitors With and Without Chemotherapy as First Line Treatment for Metastatic Non-small Cell Lung Cancer. Front. Oncol..

[113] Denkert C., von Minckwitz G., Brase J.C., Sinn B.V., Gade S., Kronenwett R., Pfitzner B.M., Salat C., Loi S., Schmitt W.D. (2015). Tumor-infiltrating lymphocytes and response to neoadjuvant chemotherapy with or without carboplatin in human epidermal growth factor receptor 2-positive and triple-negative primary breast cancers. J. Clin. Oncol..

[114] Langer C.J., Gadgeel S.M., Borghaei H., Papadimitrakopoulou V.A., Patnaik A., Powell S.F., Gentzler R.D., Martins R.G., Stevenson J.P., Jalal S.I. (2016). Carboplatin and pemetrexed with or without pembrolizumab for advanced, non-squamous non-small-cell lung cancer: A randomised, phase 2 cohort of the open-label KEYNOTE-021 study. Lancet. Oncol..

[115] Dosset M., Vargas T.R., Lagrange A., Boidot R., Vegran F., Roussey A., Chalmin F., Dondaine L., Paul C., Lauret Marie-Joseph E. (2018). PD-1/PD-L1 pathway: An adaptive immune resistance mechanism to immunogenic chemotherapy in colorectal cancer. Oncoimmunology.

[116] Voorwerk L., Slagter M., Horlings H.M., Sikorska K., van de Vijver K.K., de Maaker M., Nederlof I., Kluin R.J.C., Warren S., Ong S. (2019). Immune induction strategies in metastatic triple-negative breast cancer to enhance the sensitivity to PD-1 blockade: The TONIC trial. Nat. Med..

[117] Kroon P., Frijlink E., Iglesias-Guimarais V., Volkov A., van Buuren M.M., Schumacher T.N., Verheij M., Borst J., Verbrugge I. (2019). Radiotherapy and Cisplatin Increase Immunotherapy Efficacy by Enabling Local and Systemic Intratumoral T-cell Activity. Cancer Immunol. Res..

[118] Ock C.Y., Kim S., Keam B., Kim S., Ahn Y.O., Chung E.J., Kim J.H., Kim T.M., Kwon S.K., Jeon Y.K. (2017). Changes in programmed death-ligand 1 expression during cisplatin treatment in patients with head and neck squamous cell carcinoma. Oncotarget.

[119] Tran L., Allen C.T., Xiao R., Moore E., Davis R., Park S.J., Spielbauer K., Van Waes C., Schmitt N.C. (2017). Cisplatin Alters Antitumor Immunity and Synergizes with PD-1/PD-L1 Inhibition in Head and Neck Squamous Cell Carcinoma. Cancer Immunol. Res..

[120] Rojko L., Reiniger L., Teglasi V., Fabian K., Pipek O., Vagvolgyi A., Agocs L., Fillinger J., Kajdacsi Z., Timar J. (2018). Chemotherapy treatment is associated with altered PD-L1 expression in lung cancer patients. J. Cancer. Res. Clin. Oncol..

[121] Yan F., Pang J., Peng Y., Molina J.R., Yang P., Liu S. (2016). Elevated Cellular PD1/PD-L1 Expression Confers Acquired Resistance to Cisplatin in Small Cell Lung Cancer Cells. PLoS ONE.

[122] Fournel L., Wu Z., Stadler N., Damotte D., Lococo F., Boulle G., Segal-Bendirdjian E., Bobbio A., Icard P., Tredaniel J. (2019). Cisplatin increases PD-L1 expression and optimizes immune check-point blockade in non-small cell lung cancer. Cancer Lett..

[123] Grabosch S., Bulatovic M., Zeng F., Ma T., Zhang L., Ross M., Brozick J., Fang Y., Tseng G., Kim E. (2019). Cisplatin-induced immune modulation in ovarian cancer mouse models with distinct inflammation profiles. Oncogene.

[124] Sun L.M., Liu Y.C., Li W., Liu S., Liu H.X., Li L.W., Ma R. (2017). Nivolumab effectively inhibit platinum-resistant ovarian cancer cells via induction of cell apoptosis and inhibition of ADAM17 expression. Eur. Rev. Med. Pharm. Sci..

[125] Marcq E., Audenaerde J.R.V., Waele J., Jacobs J., Loenhout J.V., Cavents G., Pauwels P., Meerbeeck J.P.V., Smits E.L. (2019). Building a Bridge between Chemotherapy and Immunotherapy in Malignant Pleural Mesothelioma: Investigating the Effect of Chemotherapy on Immune Checkpoint Expression. Int. J. Mol. Sci..

[126] Qin X., Liu C., Zhou Y., Wang G. (2010). Cisplatin induces programmed death-1-ligand 1(PD-L1) over-expression in hepatoma H22 cells via Erk/MAPK signaling pathway. Cell Mol. Biol. Noisy Le Grand.

[127] Tsai T.F., Lin J.F., Lin Y.C., Chou K.Y., Chen H.E., Ho C.Y., Chen P.C., Hwang T.I. (2019). Cisplatin contributes to programmed death-ligand 1 expression in bladder cancer through ERK1/2-AP-1 signaling pathway. Biosci. Rep..

[128] Wakita D., Iwai T., Harada S., Suzuki M., Yamamoto K., Sugimoto M. (2019). Cisplatin Augments Antitumor T-Cell Responses Leading to a Potent Therapeutic Effect in Combination With PD-L1 Blockade. Anticancer. Res..

